# Seborrhœa and Baldness

**Published:** 1905-04-22

**Authors:** 


### SEBORRHŒA AND BALDNESS.

The relation that seborrhœa bears to the falling of the hair has been the subject of many discussions. The term "seborrhœa," to start with, is variously interpreted. To some it signifies simply an over-production of the sebaceous glands; to others a superadded inflammatory condition due to saprophytic germs; others, again, credit the germs with the production of the sebaceous excess and the inflammatory conditions together. Numerous micro-organisms have been separated from the sebaceous material, and Unna believes his morococcus, and Sabouraud considers his bacillus to be the causative agent. Pawloff has produced an alopecia in animals with the staphylococcus epidermidis albus of Welch, which also may be found in the skin secretions, while recently Whitfield has described a butyric-acid-producing bacillus from the same source.

Seborrhœa, in the sense of over-production of the sebum, may be said to be normal in certain people from childhood. Such individuals are liable to inflammatory outbreaks due, as Whitfield suggests, possibly to the fact that this secretion is a suitable culture medium for many micro-organisms and invites their active growth. It is the chronic condition of this over-production of sebum which seems associated with the falling of the hair, though how much of this is determined by the growth of any individual micro-organism is being much discussed. It seems, however, that apart from any germ-growth, activity of the sebaceous glands is somewhat antagonistic to the growth and development of hair.

At birth we frequently notice that children show marked difference in their skin secretions. Some infants have a very copious covering of the vernix caseosa; they have been described as being in a condition of serborrhœa oleosa. These children are also peculiar in that they do not show much hair at birth and are slow in growing it later. Others are "dry" babies with early production of hair. The growth of hair in the former children is at first fine and ill-nourished, and many individuals retain throughout life a proclivity towards the active production of sebaceous material.

At puberty it may be noticed that another instructive picture may be presented. The skin is extremely active and the sebaceous glands of the face often make their presence known in the unpleasant form of acne. The parts of the face most affected by this condition are naturally the "sebaceous area," that is that part of the face which is not covered with hair. It may be noticed that in young men with well-developed acne vulgaris there is a delayed production of hair on the face, and in such case the parts that should grow the whiskers and beard are also full, though not so full, of acne comedones.

Certain racial characteristics might also be cited. Negroes are "oily" men and grow hair badly. The Russian is a hairy man, but we are not in a position to state whether the sebaceous glands are therefore less active. It is a matter of common experience that Europeans in hot climates early show a tendency to become bald. Races that wear clothes lose most of the hair beneath the garments, while the sebaceous glands remain active. It would seem that exposure to cold favours the growth of hair, while exposure to warmth and sun predisposes to the poor development of hair and the greater activity of the sebaceous glands.

From these instances does it not seem possible to argue that Nature regards the production of oil and the growth of hair as alternatives in the protection of the skin? The oily secretion being the more efficient protection against heat, the hair the better protection against cold. The method by which the balance between the two is maintained is not easily determined. It is difficult to believe, when considering the way in which the hair in its development pushes its way actively between the epithelial cells, that the crusting of the sebaceous secretion about the mouths of the follicles, or the pressure of the hair in the follicle, can account for the dystrophy or atrophy of the hair. It may be that the hair bulb is relatively starved when the glands in its vicinity are making a great demand upon the blood-vessels supplying them, and *vice versâ*, that the hair bulb is more actively supplied with nourishment when the more powerful gland is resting. Perhaps, also, the action of external cold, while depressing the activity of the glands does not influence to the same extent the nutrition of the hair bulb.

If this explanation holds there is no need to inquire for any specific germ for seborrhœic alopecia. Any micro-organism which irritates the glands to secrete largely is *ipso facto* robbing the hair of its nourishment, and by the excess of oil preventing the normal effect of cold upon the glands and hair-growth. The practical outcome of this would be that the frequent removal of the oil excreted, and the acclimatisation of the scalp to changes in temperature would prove two important factors in the prevention of baldness due to seborrhœa.

